# The Surgical Clerkship Guide: A Practical Framework for Confidence and Competence

**DOI:** 10.7759/cureus.107603

**Published:** 2026-04-23

**Authors:** Vincent S Alexander, Andrew D Vogel, Maxwell J Jabaay, Michael Ernst, John Treffalls, Travis J McKenzie

**Affiliations:** 1 Department of Surgery, Alabama College of Osteopathic Medicine, Dothan, USA; 2 Department of Surgery, Mayo Clinic, Rochester, USA; 3 Department of Cardiothoracic Surgery, University of North Carolina at Chapel Hill, Chapel Hill, USA; 4 Department of Research, Alabama College of Osteopathic Medicine, Dothan, USA

**Keywords:** clinical curriculum, clinical presentations, medical education, medical student performance, surgery clerkship, surgery rotation, surgical team integration

## Abstract

Success in the surgical clerkship often depends less on prior technical skills and more on professionalism, adaptability, and the ability to integrate into a surgical team. This article provides medical students with a practical, literature-informed framework grounded in clinical experience. Drawing on published literature, the authors' experience, and structured tools, we outline key strategies for preparation, professionalism, patient care responsibilities, and operating room engagement. By applying these principles, students can reduce anxiety, maximize learning opportunities, and foster the confidence and professional identity required for future surgical training.

## Introduction

Honors-level students in a surgical clerkship stand out for their strong communication, self-reflection, clinical judgment, and ability to turn clinical data into effective care plans [1]. Prior technical skills or surgical experience are not required or expected. Instead, success depends on professionalism, preparation, observation, situational awareness, and active participation on the surgical team, rather than a student's prior operative experience [2]. Consistently showing these core skills is usually the best sign of strong clinical performance.

Even though these qualities seem clear, expectations for medical students in surgical clerkships are often not spelled out. Many students start the rotation unsure about what they should do during rounds or in the operating room (OR) [3]. This uncertainty can cause anxiety, make it harder to join the team, and lead to performance differences. It can also affect how students view surgery as a possible career.

The main goal of the surgical rotation is to provide students with exposure to the daily work of surgical care and the workflow of the OR. These settings show both sides of surgical practice and patient management, in both inpatient and outpatient care, which align with competency frameworks in medical education, including the Association of American Medical Colleges Core Entrustable Professional Activities for Entering Residency [4].

This technical report offers a step-by-step guide and workflow for approaching the surgical clerkship. The recommendations are based on commonly observed practices, teaching strategies, and structured tools that have proven effective during surgical rotations, rather than on prospective validation studies. The following sections combine evidence from surgical education literature with practical advice from the authors’ experience. When possible, statements are supported by prior studies, while other guidance draws on personal insights to help students navigate the surgical clerkship environment.

## Technical report

Key administrative details to clarify before starting include daily start times and OR schedules, call schedule and weekend responsibilities, and outpatient/inpatient versus OR balance.

Professionalism

Nguyen et al. conducted a qualitative study using semi-structured interviews and focus groups with medical students, residents, and attending surgeons. They identified five key attitudes associated with successful medical students: eagerness, humility, confidence, teamwork, and adaptability [5]. Students who proactively seek learning opportunities, remain open to constructive feedback, and demonstrate confidence in their knowledge are more likely to be entrusted with meaningful clinical responsibilities [5]. These qualities foster a positive teaching environment where faculty and residents are more inclined to invest time in teaching. Professionalism in the surgical clerkship encompasses several observable characteristics consistently associated with high-performing students.

**Figure 1:**
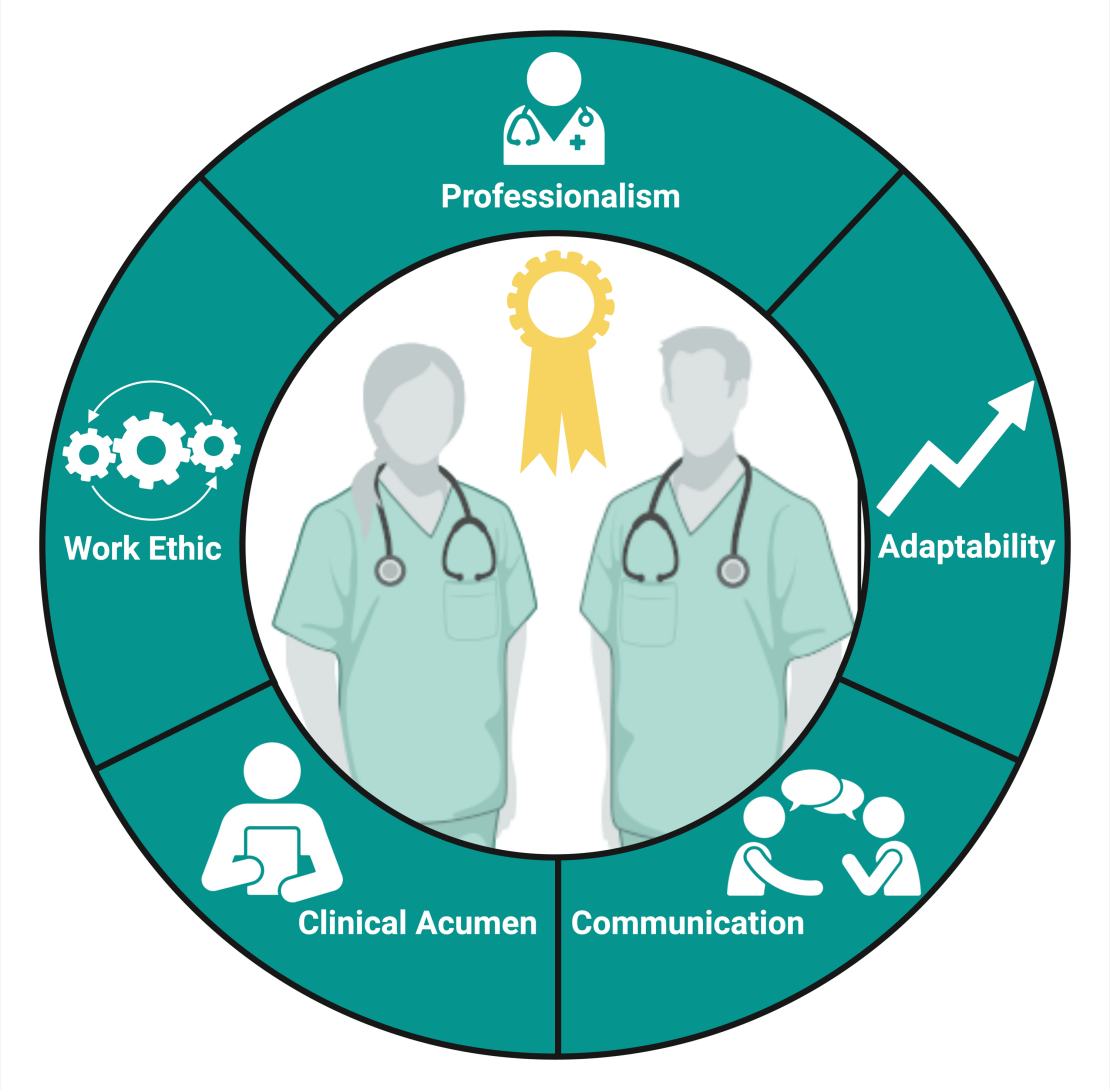
Critical characteristics of an honors-level student in a surgical clerkship A visual overview of the five foundational attributes that underpin honors-level performance in the surgical clerkship: professionalism, adaptability, communication, clinical acumen, and work ethic. Image created with BioRender.com.

Honors-level performance involves several core attributes such as strong communication skills, clinical acumen, professionalism, adaptability, and a consistent work ethic [1] (Figure 1).

Several non-negotiables are consistently seen in a strong clerkship performance. These include arriving early (at least 20 minutes) and being prepared, maintaining a positive and teachable attitude, demonstrating appropriate confidence, and treating all members of the team with respect. Learning names and remaining adaptable reinforce professionalism and facilitate an easy introduction into the surgical team.

Emotional awareness

Beyond technical knowledge, successful integration into a surgical team requires emotional awareness and situational judgment - competencies that have been recognized as important elements of effective clinical teamwork and professional development in medical training [6]. Observing the environment and team dynamics is essential during the first days of the rotation. Even if it may not be immediately apparent, the team will notice when a student is present, engaged, and making an effort to contribute meaningfully. Observing the daily workflow to anticipate needs, offering to check on post-operative patients, seeing floor patients, reviewing charts, and proactively identifying tasks show the engagement and awareness of the student.

Surgery is inherently collaborative. Students who communicate clearly, follow through on tasks, and support their colleagues are generally viewed positively by the team. When interacting with patients, demonstrating genuine empathy and taking time to explain what is happening can meaningfully improve the patient experience. Students should also feel empowered to ask questions when uncertain. Most attendings and residents appreciate a student who attempts to learn what they do not know and seeks clarification proactively.

Rounding and presentations

Depending on the institution, students may pre-round with a resident or independently before rounding with an attending physician. First, give ample time to see patients without feeling rushed. Below is a methodical and logical way to collect and present information for morning rounds:

Structured patient presentations are commonly taught using frameworks such as the SOAP (Subjective, Objective, Assessment, Plan) format. An acronym to help you remember all the information you should capture for a patient is I-OVERLAP (Table 1).

**Table 1:** Concise patient presentations using I-OVERLAP and PADBUNS NAEON, no acute events overnight

Acronym	Category	Description	Example Presentation
I	Introduction	Provide a summary of the patient, including age, medical history, type of surgery, reason for surgery, and any complications.	“Ms. Jane Doe is a 50-year-old female with a past medical history of coronary artery disease, chronic obstructive pulmonary disorder, hypertension, and hyperlipidemia who is post-op day 3 from a laparoscopic cholecystectomy for acute cholecystitis.”
O	Overnight events	List any significant overnight events affecting patient care. If none, state NAEON. Refer to Table 2 for additional information.	“There were NAEON.”
V	Vitals	Report the patient’s vital signs, including blood pressure, heart rate, oxygen saturation, and temperature. Mention interventions if needed.	“Her blood pressure ranges from 120/70-140/80. Their heart rate ranges from 70-80 beats per minute and is in sinus rhythm. They are saturating at 98% on 1-liter nasal cannula. Their temperature is 97.5 °F.”
E	(Physical) exam	Present physical exam findings, including cardiac, pulmonary, and targeted exams relevant to the surgery. Always check incision sites.	“Cardiac - regular rate and rhythm. No murmurs, gallops, or rubs heard. Pulmonary - Clear to auscultation bilaterally. Abdominal – Abdomen is flat and non-distended. Normoactive bowel sounds in all four quadrants and tympanic to percussion. Soft, non-tender, and no guarding was present on the exam. Her laparoscopic port sites look clean and are healing well.”
R	Radiology	Discuss any new radiologic imaging since the last update. Ensure you have personally reviewed the images, not just the radiology report.	“A chest X-ray from this morning demonstrates enlarged lung fields with minimal atelectasis in the lower left lung field. An abdominal X-ray demonstrates no distension of small or large bowel. These are unchanged from previous imaging.”
L	Labs	Provide lab results from the morning, comparing them to previous values to identify any trends or changes.	“Sodium 136 from 138, potassium 4 from 4.1, chloride 100 from 101, bicarbonate 25 from 25, blood urea nitrogen 10 from 11, creatinine remaining stable at 0.9, and glucose 100 from 98, white blood cell count 10.3 from 10.8, hemoglobin 10.2 from 10.4, hematocrit stable at 37, and platelet count stable at 200.”
A&P	Assessment and plan	Summarize the overall patient assessment and propose a plan addressing barriers to discharge and next steps in management.	“Ms. Doe is a 50-year-old female who is post op day #3 from a laparoscopic cholecystectomy for acute cholecystitis. There were no complications during the operation. Plan for the day is to continue oral multimodal pain control and keep her pain in between 3-4/10. Encourage ambulation today and follow physical therapy recommendations. Progress to a full diet and monitor nausea/vomiting. Wean oxygen to room air and incentivize spirometry. Confirm any barriers to discharge with case management.”

I: Introduction

This is a summary of the patient's significant medical and surgical history. Ultimately, this is telling the story of why the patient needed surgery or is being cared for by the team.

O: Overnight Events

This section includes any events that occurred overnight. One should include the most critical and pertinent things first. The overnight events are broken down into another acronym: PADBUNS (Table 2).

**Table 2:** A framework for assessing overnight events during surgical rounding PADBUNS is a mnemonic designed to guide medical students through a systematic assessment of overnight patient events during morning surgical rounds. NPO, nil per os; I/O, intake and output

Acronym	Category	Description	Example Presentation
P	Pain	Assess the patient’s pain level on a scale of 0-10, noting how well their current pain regimen is managing symptoms.	“Ms. Doe states her pain is a 5/10 and managed well on her current pain regimen.”
A	Ambulation	Determine whether the patient has been able to get out of bed, sit in a chair, walk to the hallway, or remain in bed.	“She states she walked yesterday on the floor with assistance.”
D	Diet	Describe the patient’s diet status (NPO, liquid, soft, regular, diabetic diet) and whether they are tolerating oral intake.	“She is on a liquid diet and tolerating that well with no nausea or vomiting.”
B	Bowel movement	Check if the patient has had a bowel movement or passed flatus, which is crucial for post-operative recovery.	“She notes she had her first bowel movement yesterday and has been able to urinate well.”
U	Urinary output	Report urinary output, total input/output balance, and drain/tube output. Inspect drain fluids and describe their appearance.	“Her urinary output is 3L, and her drain has produced 5mL of serosanguinous fluid over the past 24 hours. Her I/O balance is +500mL.”
N	Nausea/vomiting	Ask the patient if they have experienced nausea or vomiting, which can indicate tolerance to diet and anesthesia effects.	"Denies and nausea or vomiting last night."
S	Spirometry	Evaluate whether the patient has been using the incentive spirometer and record their achieved volumes. Encourage frequent use.	“She endorses using her spirometer every other hour.”

V: Vitals

When presenting vitals, present them as ranges from the previous 24 hours.

E: (Physical) Examination

Priorities for this will be dependent on your surgery service. For example, a colorectal service will care about gas output from an ostomy, whereas an endocrine surgery service will care about neck incision and hoarseness of the voice. A good baseline examination is to check the incision site (confirm this is okay with your resident/attending, as you do not want to remove a wound vacuum too early or a freshly placed dressing) and tailor the remaining examination to what is appropriate.

R: Radiology

Include new and updated images from the last 24 hours. Make sure to look at the imaging yourself and provide your interpretation.

L: Labs

Complete blood count and basic metabolic panel should always be included on your sheet, but one should report abnormal values or clinically actionable results. Do not just state the labs but identify the trends. Reporting abnormal labs as part of a trend is often more meaningful than isolated values.

A&P: Assessment and Plan

For the assessment, rephrase the introduction section and recap who the patient is and why they are in the hospital. For the plan, this is where most students struggle when starting clerkships. In surgery, the best thing to think about when discussing a plan is to ask, “What are the barriers to discharge for this patient?” If the presenter is asked not to include the subjective section in the presentation, this information can still be used to help develop the patient’s care plan. When formulating a plan for patients on the floor, the PADBUNS acronym should be used as a reference. For patients in the intensive care unit, a systems-based approach should be used to ensure no elements of care are overlooked, as these patients are more critically ill and more complex than those on the medical floor.

Polished presentations

The key is to be succinct in the least number of words possible and to defer erroneous words when forming your next thought. Expect to be interrupted while presenting and be ready to pick up the presentation after feedback is received from residents/attendings. Always prepare a plan if asked. A printed-out worksheet can help ask the appropriate questions during rounds and for structuring presentations (Appendix A).

Afternoon rounds

Depending on the service, afternoon rounds may or may not be planned. If they occur, the student may be required to provide updates on the patients they are following. In these situations, the plan approved during morning rounds should be reviewed, and the medical record should be checked to confirm that the planned items have been completed. Even if no afternoon rounds are scheduled, this process should still be performed. Actively following patients and monitoring their progression toward discharge is an essential component of the surgery clerkship. Students should take ownership of the patients they are responsible for and see them at least in the morning and the afternoon.

The operating room

**Figure 2:**
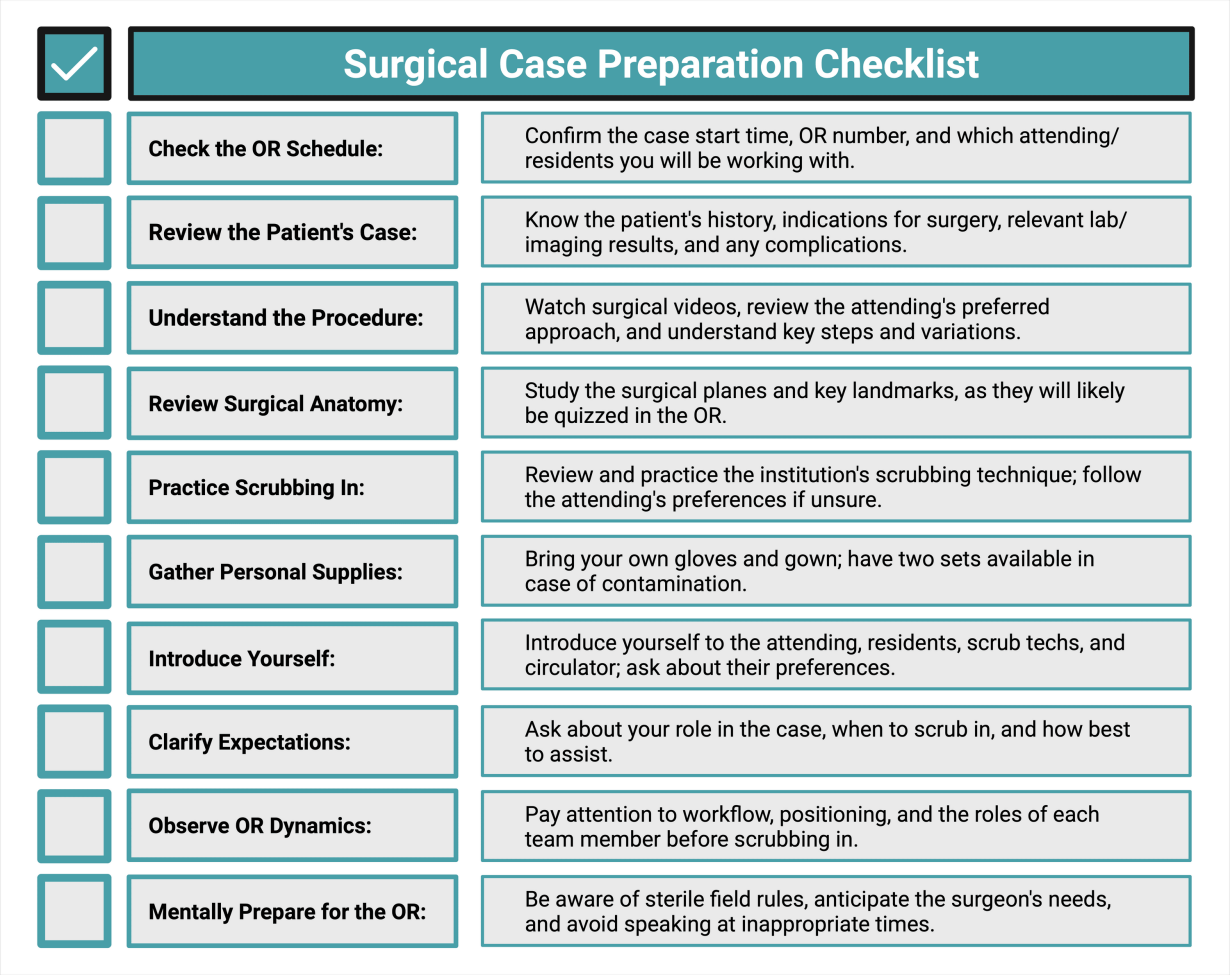
Key preparation tasks for students to ensure readiness for the operating room A structured guide emphasizing the essential tasks medical students should complete before entering the operating room. Key preparations include confirming logistical details, reviewing the patient’s case and relevant surgical anatomy, practicing institution-specific scrubbing techniques, and gathering personal supplies. Image created with BioRender.com.

Students new to the OR may benefit from reviewing proper gowning and scrubbing procedures before their first case. Active practice is necessary. If it is their first time working with a particular attending or resident, they should volunteer to scrub in alongside them to ensure that they are following preferred practices. Once familiar with the service, students may scrub in ahead of the attending or resident but should always confirm that it is appropriate to do so beforehand (Figure 2).

Tip

Students should expect to grab their own gloves and gown, two sets of each, but always ask first. They should never assume it is acceptable to place a sterile gown or gloves on a sterile surface or the Mayo stand without permission. It is important to ask the scrub technician before doing so. Additionally, nothing should be removed from the sterile field without explicit permission.

Anticipate next steps

Anticipating the next steps in surgery can be challenging for students new to the OR, especially since preferences vary between attendings. The key to developing this skill is active observation and attentiveness. Pay close attention to how the attending and first assistant work together. Understanding these nuances allows you to better anticipate needs. Outside the OR, it is beneficial to refer to reputable surgical videos available on online platforms for the cases planned the next day.

Practice suturing

Confidence grows through consistent, deliberate practice. Use available reputable resources to learn suture techniques and build skills outside the OR. Practice both one-handed and two-handed knots regularly. A stepwise approach is recommended: begin with shoelaces or rope to focus on form, progress to surgical gloves, and, finally, practice with wet suture material. Familiarity with these motions reduces anxiety and fosters comfort in the OR. Before attempting suturing, handle basic instruments (needle drivers, Adson forceps, DeBakey forceps, Metzenbaum scissors, and Mayo scissors) with confidence and skill. Practice proper grips, hand positioning, and efficient tool passing to improve accuracy and speed. Practicing on suture pads, then transitioning to chicken thighs or feet.

## Discussion

Prior work has demonstrated subtle but meaningful discrepancies between resident and attending expectations regarding student responsibilities in rounding, clinic participation, OR engagement, and professionalism. Even when general agreement exists, variation in specific performance standards can create uncertainty for learners. Such ambiguity can undermine confidence, impede integration into the surgical team, and contribute to inconsistent evaluations. Establishing explicit expectations has been shown to improve student understanding of roles and grading processes [3].

A single-center study that surveyed medical students found that students who achieved honors were more likely to have higher United States Medical Licensing Examination Step 1 scores, strong grit, and an individual-based learning style [7]. While academic ability played a role, students who demonstrated perseverance and adaptability, hallmarks of grit, were also more likely to excel [7,8]. It is important to note that the defining characteristics do not include technical skills or prior surgical experience. Student success during the surgical clerkship is driven primarily by professionalism, preparation, situational awareness, and effective involvement with the surgical team, rather than preexisting technical skill [1]. Notably, objective examination performance does not necessarily reflect clinical excellence. Saberi et al., in a retrospective cohort study of 625 medical students at a single institution from 2016 to 2020, demonstrated that clinical performance during the third-year surgery clerkship showed only a negligible correlation with NBME shelf, oral examination, and quiz scores [9].

By operationalizing what can constitute an efficient and informative presentation, effective postoperative assessments, and appropriate intraoperative conduct, students may be better positioned to demonstrate clinical acumen and reliability. This transition from tacit norms to more explicit standards promotes consistency in performance and may mitigate disparities rooted in informal knowledge transmission, a phenomenon in medical education known as the "hidden curriculum", the informal, unspoken norms that shape learning outside formal instruction [10].

Several limitations should be acknowledged. First, the guide is consensus-based and narrative in design, without prospective data demonstrating improved clerkship performance, examination outcomes, or residency match success. Its recommendations are practical and experience-informed but have not been formally validated across institutions. Conceptual discussions of professionalism, emotional awareness, and predictors of successful clerkship performance draw primarily from published literature, while the sections outlining administrative preparation, rounding strategies, OR conduct, and deliberate technical practice represent experience-informed recommendations intended to translate these principles into actionable behaviors for students. Second, generalizability may be limited. Clerkship structure, grading systems, operative culture, and resident expectations vary widely between programs. The frameworks described will likely require adaptation to local workflows and institutional norms. Third, while the guide emphasizes professionalism, preparation, and structured presentations, student performance may also be influenced by factors not directly addressed, including variability in teaching quality, evaluative subjectivity, workload intensity, and implicit bias [11]. Fourth, recommendations for deliberate practice and operative preparation assume access to time and resources that may not be equally available to all students. Finally, the guide focuses on optimizing performance, which, if not contextualized appropriately, may contribute to performance pressure in an already high-stakes environment.

## Conclusions

Students must recognize that their presence in the OR may occasionally slow the workflow, a natural and expected part of the learning process. Surgical teams understand that students are there to gain experience and develop their skills rather than perform at a resident’s level from the outset. By staying present in the moment for every teaching opportunity and focusing on the learning process rather than external pressure from others, students can transform what might feel like a stressful situation into a valuable educational experience. Over time, with practice and patience, confidence and efficiency will naturally follow.

The structured tools presented in this guide, including the I-OVERLAP patient presentation framework, PADBUNS rounding checklist, and OR preparation strategies, provide practical approaches that students can immediately apply during their surgical clerkship.

## Figures and Tables

**Figure 3:**
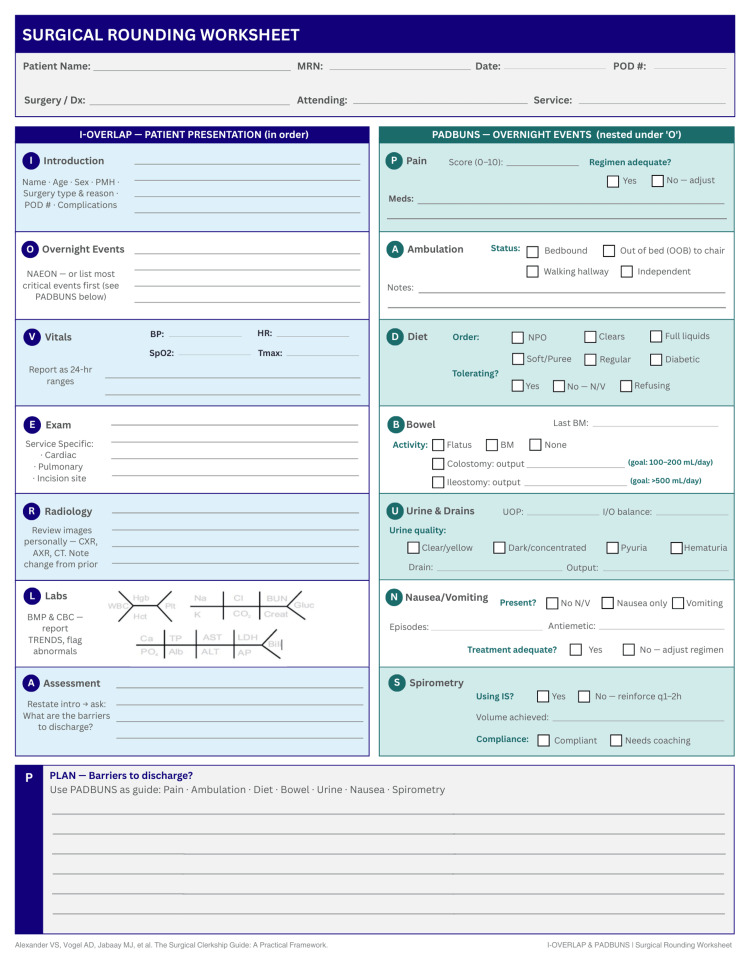
Printable worksheet for presentations and rounds

## Appendices

Appendix A 
